# Involvement of Autophagy in Rat Tail Static Compression-Induced Intervertebral Disc Degeneration and Notochordal Cell Disappearance

**DOI:** 10.3390/ijms22115648

**Published:** 2021-05-26

**Authors:** Takashi Yurube, Hiroaki Hirata, Masaaki Ito, Yoshiki Terashima, Yuji Kakiuchi, Ryosuke Kuroda, Kenichiro Kakutani

**Affiliations:** Department of Orthopaedic Surgery, Kobe University Graduate School of Medicine, 7-5-1 Kusunoki-cho, Chuo-ku, Kobe 650-0017, Japan; hiroaki928@hotmail.com (H.H.); maito28710@yahoo.co.jp (M.I.); y.teratera0831@gmail.com (Y.T.); yuji_uz_7@yahoo.co.jp (Y.K.); kurodar@med.kobe-u.ac.jp (R.K.); kakutani@med.kobe-u.ac.jp (K.K.)

**Keywords:** intervertebral disc degeneration, nucleus pulposus, notochordal cells, autophagy, apoptosis, nutrient deprivation, static compression loading, animal model, low back pain, spine

## Abstract

The intervertebral disc is the largest avascular low-nutrient organ in the body. Thus, resident cells may utilize autophagy, a stress-response survival mechanism, by self-digesting and recycling damaged components. Our objective was to elucidate the involvement of autophagy in rat experimental disc degeneration. In vitro, the comparison between human and rat disc nucleus pulposus (NP) and annulus fibrosus (AF) cells found increased autophagic flux under serum deprivation rather in humans than in rats and in NP cells than in AF cells of rats (*n* = 6). In vivo, time-course Western blotting showed more distinct basal autophagy in rat tail disc NP tissues than in AF tissues; however, both decreased under sustained static compression (*n* = 24). Then, immunohistochemistry displayed abundant autophagy-related protein expression in large vacuolated disc NP notochordal cells of sham rats. Under temporary static compression (*n* = 18), multi-color immunofluorescence further identified rapidly decreased brachyury-positive notochordal cells with robust expression of autophagic microtubule-associated protein 1 light chain 3 (LC3) and transiently increased brachyury-negative non-notochordal cells with weaker LC3 expression. Notably, terminal deoxynucleotidyl transferase dUTP nick end labeling-positive apoptotic death was predominant in brachyury-negative non-notochordal cells. Based on the observed notochordal cell autophagy impairment and non-notochordal cell apoptosis induction under unphysiological mechanical loading, further investigation is warranted to clarify possible autophagy-induced protection against notochordal cell disappearance, the earliest sign of disc degeneration, through limiting apoptosis.

## 1. Introduction

Back pain is a global health problem with a high morbidity of 70%–85% in the lifetime [1] and socioeconomic burden of ≈$100 billion/year in the US [2]. The cause of back pain is multifactorial. However, as a large-scale twin study showed [3], intervertebral disc degeneration plays an important role in back pain. Furthermore, disc degeneration is associated with serious neurological complications including radiculopathy, myelopathy, and paralysis [4]. Disc degeneration appears with age in approximately 40% of people under 30 years and 90% of those over 55 years [5], leading to impaired daily activities of the elderly [6]. Therefore, there is a great need to understand how aging affects the physiology of the intervertebral disc.

The intervertebral disc has a complex structure with the central, gelatinous nucleus pulposus (NP) encapsulated by the collagenous, laminar annulus fibrosus (AF) and cartilage endplates [4], providing the function of load, shock absorption, and movement of the spine [7]. In development, while the AF arises from the mesenchyme [8,9], the NP originates from the notochord [10]. Disc NP notochordal cells exist only the first ≈10 years of human life [8], subsequently during degeneration replaced by non-notochordal chondrocyte-like cells, the phenotype of which has more recently been identified as the representation of a terminal differentiation stage of notochordal cells [11]. Morphological and biochemical disc degeneration starts from early childhood [12,13], which is generally more severe in the NP than in the AF [13]. Apoptosis, programmed cell death, also increases in ages 11–16 years, which is associated with notochordal cell disappearance and chondrocyte proliferation [13]. A notably high incidence of apoptosis has also been reported in human discs with aging and degeneration [14]. These lines of evidence suggest a possible link between apoptosis and notochordal cell disappearance in the pathogenesis of age-related disc degeneration [8].

The intervertebral disc is the largest avascular, immune-privileged, low-nutrient organ in the body [15]. Particularly in the disc central NP compared to the peripheral AF, cells depend on the diffusion from blood vessels at the disc margins to obtain nutrients. Therefore, decreased blood supply, subchondral bone sclerosis, and endplate calcification, all of which occur with mechanical stress, injury, smoking, and then aging, can reduce transport of nutrients to the disc [15]. This additional loss of nutrient supply is a suspected initiator of intervertebral disc degeneration [15].

Autophagy, the intracellular process by which cells degrade and recycle their own damaged components, is an important cell survival mechanism to sustain metabolism and to prevent the accumulation of damaged, toxic proteins and organelles under stress, primarily nutrient deprivation [16,17,18]. Autophagy involves autophagy-related (Atg) genes and proteins [16,17,18]. Under physiological conditions, basal autophagy serves as a quality-control mechanism for cellular renovation and homeostasis with relatively low expression levels of Atg proteins [17]. Under stress conditions, Atg proteins are activated for the formation, maturation, and degradation of the autophagosome that captures damaged organelles, misfolded proteins, and invading microorganisms in induced autophagy [16], ultimately leading to the release and reutilization of its constituents by the fusion with the lysosome [16,17]. Monitoring of this dynamic, multistep process, called “autophagic flux [18]”, is essential to understand the execution of autophagy.

An extremely harsh environment of low nutrition, pH, and oxygen concentration in the intervertebral disc [15] raised a question of whether resident cells utilize autophagy to cope with the physiologically stressful conditions. Thus, we hypothesized that autophagy would be pivotal in maintaining disc health and homeostasis, especially for notochordal cells originally living in the avascular, low-nutrient central NP. In fact, disc cellular autophagy [19,20] with related intracellular signaling networks [21,22] has increased interest, as summarized by a review [23]. However, few studies have focused on the relationship of notochordal cell disappearance with autophagy as well as with apoptosis. Therefore, in vitro and in vivo studies were designed to elucidate the involvement and roles of autophagy in experimental disc degeneration and notochordal cell disappearance using a mechanical loading-induced animal model. We selected a rat tail static compression model [24,25,26,27,28,29] mimicking time-course radiological, histological, and biochemical disc degeneration [24,25,28,29] with extracellular matrix degradation [24,26,28], apoptotic cell death [27,28], and notochordal cell disappearance [27,28], all of which should prove useful for understanding of age-related changes in intervertebral disc degeneration.

## 2. Results

### 2.1. Autophagy in Human Degenerated and Rat Non-Degenerated Disc NP and AF Cells

To understand the variation in autophagic response by the cell type, degeneration severity, and species, we performed in vitro experiments using human degenerated and rat non-degenerated disc NP and AF cells. Levels of serum deprivation-induced autophagy were compared between lumbar disc NP and AF cells from adult humans surgically obtained and young healthy rats (*n* = 6/species, cell type, and experimental condition).

**Figure 1:**
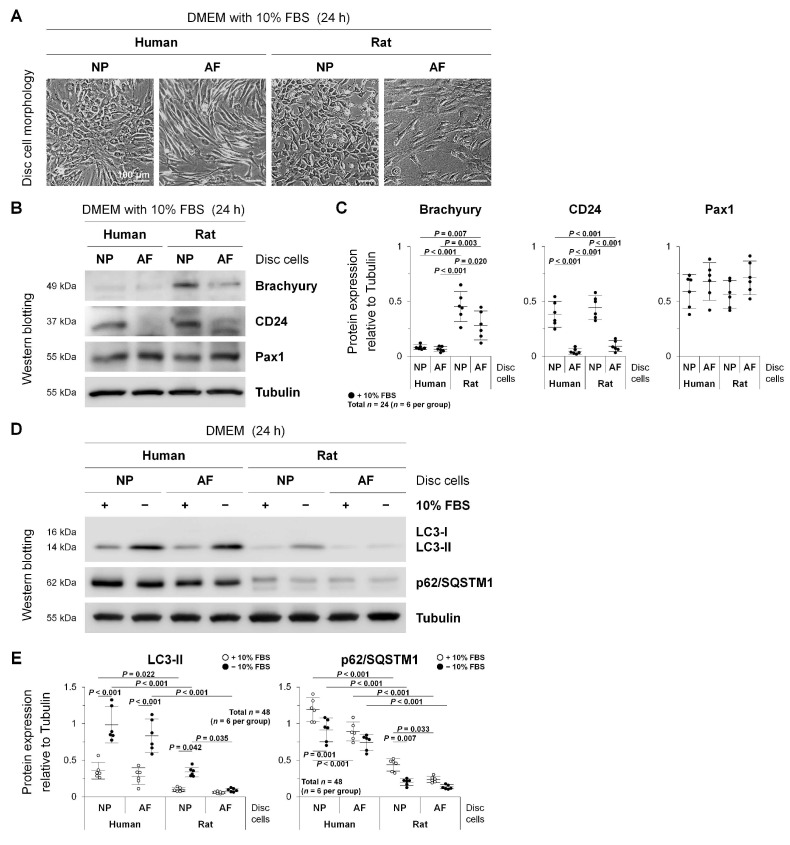
Autophagy in human degenerated and rat non-degenerated disc NP and AF cells. (**A**) Morphological appearance of human and rat lumbar disc NP and AF cells after 24 h culture in DMEM with 10% FBS. Cellular images (human NP and AF cells from the L4–L5 disc with the Pfirrmann grade 3 in a 64-year-old woman) are representative of experiments with similar results (*n* = 6). (**B**) Western blotting for NP notochordal markers brachyury and CD24 and NP and AF marker Pax1 in total protein extracts from human and rat lumbar disc NP and AF cells after 24 h culture in DMEM with 10% FBS. Tubulin was used as a loading control. Immunoblots shown (human NP and AF cells from the L4–L5 disc with the Pfirrmann grade 3 in a 64-year-old woman) are representative of experiments with similar results (*n* = 6). (**C**) Changes in protein expression of brachyury, CD24, and Pax1 relative to tubulin in human and rat lumbar disc NP and AF cell protein extracts. Data are the mean ± 95% CI. Two-way ANOVA with the Tukey–Kramer post-hoc test was used (*n* = 6). (**D**) Western blotting for autophagy marker LC3, autophagy substrate p62/SQSTM1, and loading control tubulin in total protein extracts from human and rat lumbar disc NP and AF cells after 24 h culture in DMEM with and without 10% FBS for serum deprivation. Immunoblots shown (human NP and AF cells from the L4–L5 disc with the Pfirrmann grade 3 in a 64-year-old woman) are representative of experiments with similar results (*n* = 6). (**E**) Changes in protein expression of LC3-II and p62/SQSTM1 relative to tubulin in human and rat lumbar disc NP and AF cell protein extracts. Data are the mean ± 95% CI. Two-way ANOVA with the Tukey–Kramer post-hoc test was used (*n* = 6).

Morphologically, while human and rat disc NP cells were chondrocyte-like and cobblestone-shaped, AF cells were fibroblast-like and spindle-shaped, suggesting a successful tissue acquisition and cell identification (Figure 1A).

As notochord-related disc NP markers, brachyury and CD24 were selected based on the recommendations [11,30,31]. In addition, paired box 1 (Pax1) is relatively disc NP-specific [31,32] and AF-specific [33]. Western blotting showed that CD24 was useful to distinguish NP cells from AF cells (*p* < 0.001, humans and rats), whereas brachyury was more distinct in rats than in humans (*p* < 0.001, NP; *p* = 0.003, AF), indicating the higher specificity for the notochord (Figure 1B,C). Meanwhile, Pax1 expression was common between human and rat disc NP and AF cells; consequently, the specificity of Pax1 was not so high between the NP and AF (Figure 1B,C). These findings support an acceptable harvest of disc region-specific cells.

The microtubule-associated protein 1 light chain 3 (LC3) (mammalian Atg8 homolog) is a ubiquitin-like protein, and its phosphatidylethanolamine-conjugated form, LC3-II (unlike its cytosolic form, LC3-I), is the only protein marker [18] reliably associated with the completed autophagosome [34]. The p62/sequestosome 1 (p62/SQSTM1) is a ubiquitin-binding protein serving as a link between LC3 and ubiquitinated substrates, and p62/SQSTM1 and p62/SQSTM1-bound polyubiquitinated proteins are selectively degraded after the incorporation into the autophagosome [35], negatively correlating with autophagosome degradation [18]. These were used to assess autophagic flux. Western blotting demonstrated that serum withdrawal increased LC3-I to LC3-II conversion (*p* < 0.001, human NP and AF; *p* = 0.042, rat NP) and decreased p62/SQSTM1 expression (*p* = 0.001, human NP; *p* = 0.007, rat NP), both indicating enhanced autophagy; therefore, autophagic changes were common between human and rat discs and between NP and AF cells (Figure 1D,E). Then, serum-supplemented basal levels of LC3-II (*p* = 0.022, NP) and p62/SQSTM1 (*p* < 0.001, NP and AF) and serum-deprived induced levels of LC3-II (*p* < 0.001, NP and AF) and p62/SQSTM1 (*p* < 0.001, NP and AF) were markedly higher in humans than in rats, possibly because of elevated autophagy in human surgical disc specimens at disease states (Figure 1D,E). The difference in autophagy induction was not statistically significant between human disc NP and AF cells; however, serum starvation-induced LC3-II expression was significantly higher in rat disc NP cells than in AF cells (*p* = 0.035), which was possibly due to abundant notochordal cells of rat disc NP regions (Figure 1D,E). These findings indicate that autophagic response would be affected by the cell type, degeneration severity, and species.

### 2.2. Autophagy in Rat Tail Sustained Static Compression-Induced Disc Degeneration

**Figure 2:**
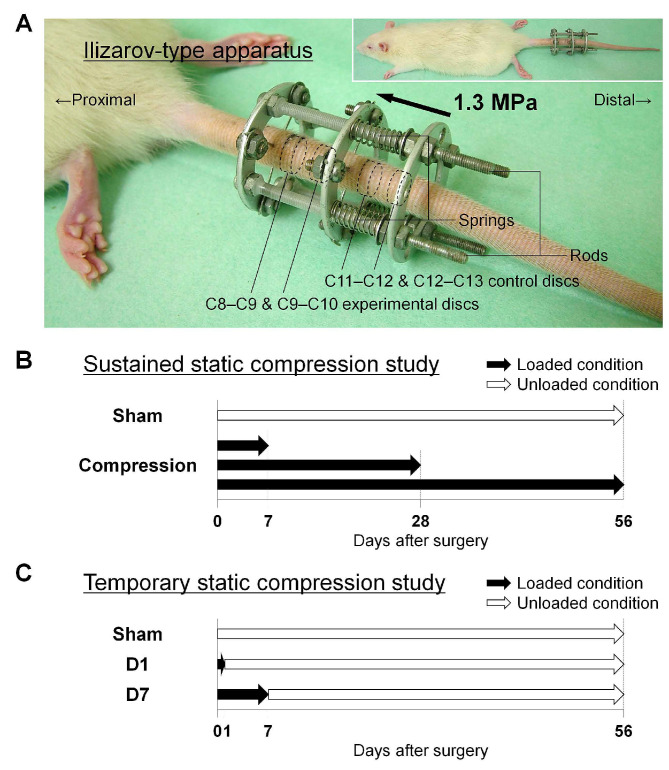
Rat tail sustained and temporary static compression model study. (**A**) Whole and close-up views of a rat tail instrumented with an Ilizarov-type loading device with springs and rods between the 8th and 10th coccygeal (C) vertebrae to produce axial force at 1.3 MPa. Following compression, C8–C9 and C9–C10 experimental discs and C11–C12 and C12–C13 control discs were used for evaluation. (**B**) Schematic illustration regarding the loading protocol of 0-day (sham, unloaded for 56 days), 7-day, 28-day, and 56-day sustained static compression. (**C**) Schematic illustration regarding the loading protocol of 0-day (sham, unloaded for 56 days), 1-day (D1, loaded for the first 24 h and subsequently unloaded for 55 days), and 7-day (D7, loaded for the initial 7 days and subsequently unloaded for 49 days) temporary static compression.

To clarify the involvement of autophagy during intervertebral disc degeneration, we performed in vivo experiments using a rat tail sustained static compression model previously established (Figure 2A) [24,25,26,27]. In this model, rat tails were instrumented with an Ilizarov-type apparatus with springs [36], and axial force was applied to produce a calculated compressive stress of 1.3 MPa [24,25,26,27]. This pressure, corresponding closely to transient disc loading force produced by lifting a moderate weight in the human lumbar spine, is shown to induce morphological and biochemical disc degeneration [37,38]. Discs were randomly loaded for up to 56 days after surgery (7-day, 28-day, or 56-day compression) or unloaded for 56 days after sham surgery (0-day compression). Following compression, experimental and control discs were analyzed by Western blotting and immunohistochemistry (*n* = 6/time point and experimental condition) (Figure 2B). All rats underwent surgery without any problems and gained body weight throughout the experiment (452–509 g at surgery and 543–614 g at 56 days). All springs maintained their compressive length without instrument failure and fully recovered immediately after release, indicating sustained axial loading. There were no signs of infection, skin necrosis, or neurological problems.

**Figure 3:**
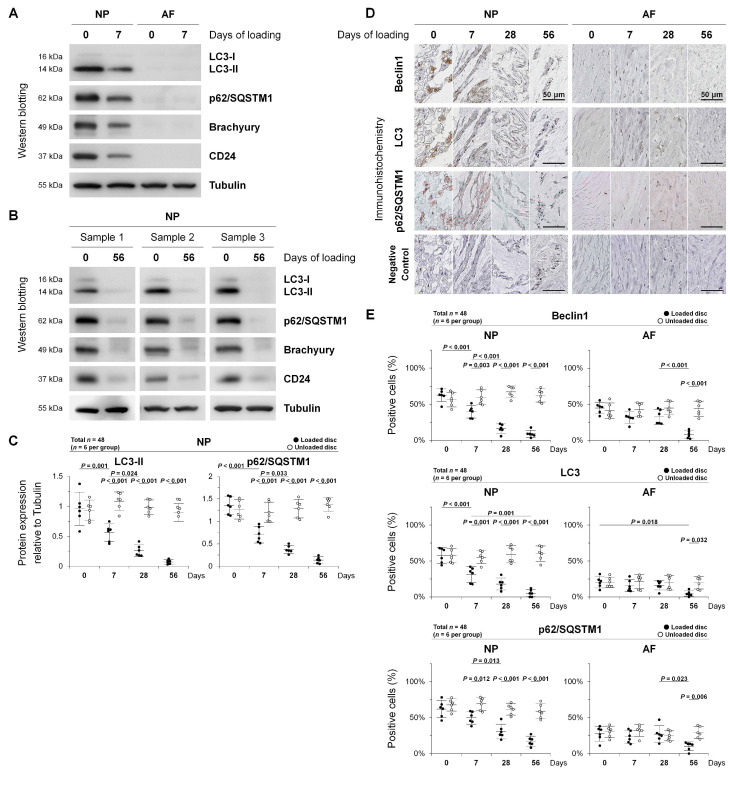
Autophagy in rat tail sustained static compression-induced disc degeneration. (**A**) Time-course Western blotting for autophagy marker LC3, autophagy substrate p62/SQSTM1, and NP notochordal markers brachyury and CD24 in total protein extracts from rat tail loaded disc NP and AF tissues at 0-day (sham) and 7-day sustained static compression. Tubulin was used as a loading control. Immunoblots shown are representative of experiments with similar results (*n* = 6). (**B**) Time-course Western blotting for autophagic LC3 and p62/SQSTM1 and loading control tubulin in rat tail loaded disc NP tissue protein extracts at 0-day (sham) and 56-day sustained static compression. Immunoblots shown are three representatives of experiments with similar results (*n* = 6). (**C**) Changes in protein expression of LC3-II and p62/SQSTM1 relative to tubulin in rat tail loaded and unloaded NP tissue protein extracts at 0-day (sham), 7-day, 28-day, and 56-day sustained static compression. Data are the mean ± 95% CI. Two-way ANOVA with the Tukey–Kramer post-hoc test was used (*n* = 6). (**D**) Time-course immunohistochemistry for autophagy-related beclin1, LC3, p62/SQSTM1, and negative control in rat tail loaded disc NP and AF sections at 0-day (sham), 7-day, 28-day, and 56-day sustained static compression. Immunoimages shown are representative of experiments with similar results (*n* = 6). (**E**) Changes in the percentage of beclin1-positive cells, LC3-positive cells, and p62/SQSTM1-positive cells in rat tail loaded and unloaded disc NP and AF sections at 0-day (sham), 7-day, 28-day, and 56-day sustained static compression. Data are the mean ± 95% CI. Two-way ANOVA with the Tukey–Kramer post-hoc test was used (*n* = 6).

To monitor autophagic flux in disc NP notochordal cells, we conducted Western blotting for autophagy-related LC3 and p62/SQSTM1 and NP notochord-related brachyury and CD24. At 0 day of sham loading, much more abundant LC3-II and p62/SQSTM1 expression was observed in the disc NP than in the AF (Figure 3A). Successful disc region-specific tissue acquisition was confirmed by NP notochord-related brachyury and CD24 expression (Figure 3A,B). At 7 days of loading, marked decreases in the expression of LC3-II (*p* = 0.001) and p62/SQSTM1 (*p* < 0.001) as well as brachyury and CD24 were shown in the disc NP (Figure 3A,C). At 56 days of loading, these decreases in autophagy-related and NP notochord-related proteins were time-dependent in damaged NP tissues (both *p* < 0.001), while it was difficult to collect enough proteins in damaged AF tissues (Figure 3B,C). Time-course analysis identified reduced levels of autophagy at least in severely degenerated discs under unphysiological chronic mechanical loading.

Then, we performed immunohistochemistry for autophagy-related LC3, p62/SQSTM1, and also beclin1 (mammalian Atg6 homolog), acting as a key autophagosome initiation regulator [18,39]. Positive controls showed strong staining, while IgG controls were negative. Beclin1, LC3, and p62/SQSTM1 showed cytoplasmic localization. Notably, marked immunoreactivity for these autophagy-related proteins was observed in rat disc NP cells—particularly large, vacuolated notochordal cells (Figure 3D). Time-course observation exhibited trends toward decrease throughout the loading duration in immunopositivity for beclin1, LC3, and p62/SQSTM1 in the disc NP (all *p* < 0.001) and AF (*p* = <0.001–0.018) (Figure 3D,E). These findings indicate decreased disc cellular autophagy depending on the degeneration severity, which is remarkable in disc NP notochordal cells.

### 2.3. Autophagy, Apoptosis, and Notochordal Cell Disappearance in Rat Tail Temporary Static Compression-Induced Disc Degeneration

To disclose possible roles of autophagy and apoptosis in disc NP notochordal cells, we performed additional in vivo experiments using a rat tail temporary static compression model (Figure 2A) [28,29]. Compared to sustained compression-induced expedited disc degeneration [24,25,26,27], temporary compression is more suited to study earlier events of degeneration, including notochodal cell disappearance [28,29]. Rats were randomly divided into three groups based on the loading duration: sham—unloaded throughout, D1—unloaded after the first 24 h compression, and D7—unloaded after the initial 7-day compression. At 56 days after surgery, experimental and control discs were analyzed by histomorphology and immunofluorescence (*n* = 6/experimental condition) (Figure 2C). All animals tolerated surgery well and subsequent growth without infection, skin necrosis, neurological problems, or instrument failure (435–495 g at surgery and 557–650 g at 56 days).

**Figure 4:**
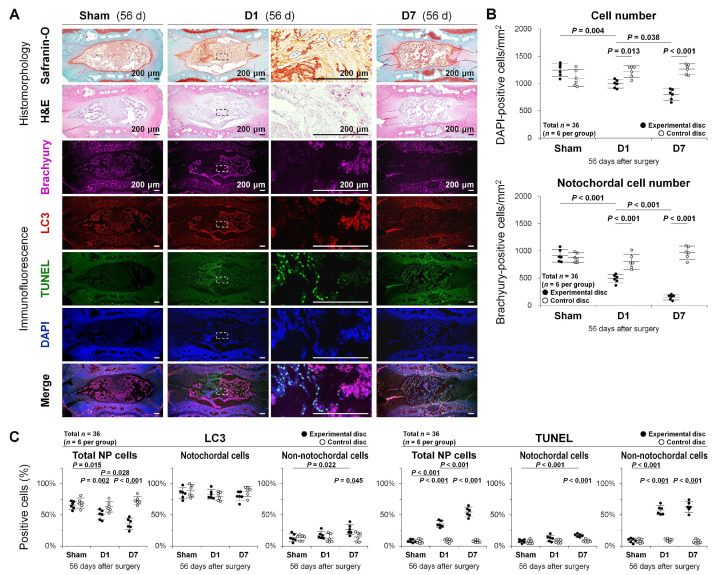
Autophagy, apoptosis, and notochordal cell disappearance in rat tail temporary static compression-induced disc degeneration. (**A**) Safranin-O, fast green, and hematoxylin staining, H&E staining, and multi-color immunofluorescence for NP notochordal brachyury (purple), autophagic LC3 (red), apoptotic TUNEL (green), nuclear DAPI (blue), and merged signals in rat tail experimental disc NP sections at 56 days of 0-day (sham, unloaded throughout), 1-day (D1, loaded for the first 24 h and unloaded later), and 7-day (D7, loaded for the initial 7 days and unloaded later) temporary static compression. Immunoimages shown are representative of experiments with similar results (*n* = 6). (**B**) Changes in the number of DAPI-positive total cells and brachyury-positive notochordal cells in rat tail experimental and control disc NP sections at 56 days of 0-day (sham), 1-day (D1), and 7-day (D7) temporary static compression. Data are the mean ± 95% CI. Two-way ANOVA with the Tukey–Kramer post-hoc test was used (*n* = 6). (**C**) Changes in the percentage of LC3-positive cells and TUNEL-positive cells relative to DAPI-positive total cells, brachyury-positive notochordal cells, and brachyury-negative non-notochordal cells in rat tail experimental and control disc NP sections at 56 days of 0-day (sham), 1-day (D1), and 7-day (D7) temporary static compression. Data are the mean ± 95% CI. Two-way ANOVA with the Tukey–Kramer post-hoc test was used (*n* = 6).

Safranin-O, fast green, and hematoxylin staining and hematoxylin and eosin (H&E) staining were performed to visualize disc cellular constituents and proteoglycan distribution. In response to temporary static compression, increased degenerative signs including morphological disappearance of large, vacuolated NP notochordal cells, and altered disc matrix components were shown (Figure 4A).

Multi-color immunofluorescence for NP notochord-related brachyury, autophagy-related LC3, apoptosis-related terminal deoxynucleotidyl transferase dUTP nick end labeling (TUNEL) for fragmented DNA detection [40], and 4’,6-diamidino-2-phenylindole (DAPI) for counterstaining was further performed to describe the population, distribution, and characteristics of disc NP cells. The number of DAPI-positive total disc NP cells decreased with the compression period (sham, D1, D7: 100.0%, 79.8%, 64.0%; *p* = <0.001–0.038) (Figure 4A,B). Then, the number of immunopositive cells for brachyury, identified as notochordal cells (showing nuclear localization), decreased more rapidly (sham, D1, D7: 73.2%/total (100.0%), 49.6%/total (54.0%), 18.4%/total (16.1%); all *p* < 0.001) (Figure 4A,B). The percentage of immunopositive cells for LC3 (showing cytoplasmic localization) in total cells also decreased with the loading period (*p* = <0.001–0.028). Autophagic LC3 staining was predominant in brachyury-positive notochordal cells, which was low in brachyury-negative non-notochordal cells of the sham and D1 but increased in the D7 (*p* < 0.001) (Figure 4A,C). Meanwhile, the percentage of TUNEL-positive cells (showing nuclear localization) in total NP cells increased in parallel with the loading duration (all *p* < 0.001), which increased in non-notochordal cells of the D1 and D7 (both *p* < 0.001) but only in notochordal cells of the D7 (*p* < 0.001). Apoptotic TUNEL staining was positive in a few cells of the sham, which was however predominant in brachyury-negative non-notochordal cells of the D1 and D7 (Figure 4A,C). Positivity in total NP cells was not from the simple addition of positivity in notochordal and non-notochordal cells due to a drastic loss of notochordal cells (Figure 4C). These findings indicate temporary static compression-induced rapid decreases in notochordal cells with robust autophagic protein expression and transient increases in non-notochordal cells with frequent apoptotic DNA fragmentation.

## 3. Discussion

This study describes an intensive involvement of autophagy and its possible anti-apoptotic and notochordal phenotype-maintaining roles in disc NP notochordal cells. In vitro, serum starvation-induced autophagy was common in human and rat disc cells but greater in NP cells than in AF cells of rats. In vivo, autophagy-related protein expression was markedly higher in the rat disc NP than in the AF—notably in notochordal cells, which both declined by sustained static compression. Furthermore, temporary static compression decreased notochordal cells with abundant autophagic proteins and increased non-notochordal cells with limited autophagy but promoted apoptosis. These findings indicate the substantial involvement of autophagy in intervertebral disc degeneration and notochordal cell disappearance, suggesting its role in maintaining disc health through apoptosis inhibition.

In vitro, serum deprivation-induced autophagy in disc NP and AF cells is consistent with prior reports [19,20,23,41]. To mimic endplate closure and subsequent cut off of serum supply, in vitro responses to limited nutrition were observed. However, no studies have directly compared autophagy levels between human and animal discs and between NP and AF cells. Morphological appearances of disc NP and AF cells are in agreement with reported evidence [4]. In the comparison between rat disc NP and AF cells, the presence of notochordal cells in the NP is the biggest difference [9], thereby suggesting a distinct involvement of autophagy in NP notochordal cells. However, in the comparison between human and rat discs, NP-cell and AF-cell autophagy levels were higher in humans than in rats. This is controversial, as adult human discs lose the notochordal phenotype [8], which could be interpreted by surgical acquisition of human specimens undergoing diverse stresses including degeneration, inflammation, disease, and intraoperative invasion. Autophagy is a stress-response machinery [16]. In human chondrocytes, compared to femoral head cartilage surgically collected from fracture patients (non-degenerated), LC3 expression increased in lateral femoral condyle cartilage (mildly degenerated) and decreased in medial femoral condyle cartilage (severely degenerated) harvested from varus-type knee osteoarthritis patients [42]. In human disc cells, *ATG* gene expression was up-regulated in Pfirrmann [43] degeneration grades 4–5 discs, compared to grades 1–3 discs [44]; however, autophagic flux should be monitored at either the protein or organelle levels [18]. A Western blotting study of human degenerative disc tissues surgically collected found peaked LC3-II protein expression in Pfirrmann [43] grade 3 compared to grades 2, 4, and 5 as well as severity-dependent increases in p62/SQSTM1 expression [45]. We also studied autophagy-related ATG5, LC3-II, and p62/SQSTM1 protein expression in human lumbar disc surgical specimens and observed age-dependent decreases in LC3-II with unaffected ATG5 and p62/SQSTM1, suggesting impaired autophagy at least in older-aged discs [20]. Further investigation is necessary. Nevertheless, this study is the first to demonstrate that autophagy is common among human and rat disc NP and AF cells, but their autophagy levels can vary by the cell type, degeneration severity, species, and residual notochordal phenotype.

In vivo, sustained static compression-induced decreases in rat disc LC3-II and p62/SQSTM1 are compatible with a prior mouse knee osteoarthritis study, showing progressive decreases in Atg protein expression after transection of the medial meniscotibial and collateral ligaments [46]. A drastic shift toward catabolism in experimentally expedited disc and cartilage degeneration would persistently diminish autophagy possibly due to disturbed cellular stress-response functions. The current study findings support that unphysiological chronic mechanical stress impairs autophagic flux at least in severely degenerated discs.

In sham rats, autophagy was enhanced with abundant autophagy-related proteins in healthy disc NP notochordal cells. Moreover, under static compression, the expression of autophagy-related proteins and notochordal markers decreased in parallel. In spontaneous aging studies, Atg protein expression decreased in the mouse knee cartilage [46] but increased in the rat disc NP [47]. This difference can also be explained by disc NP notochordal cells. Age-related disc autophagy induction may be requested to renovate notochordal cells because of their markedly slow proliferation [48]. Rodents preserve notochordal cells in the disc NP throughout the lifetime [9]. This limits relevance to the human situation [8]; however, rodent models provide insight into notochordal cell-related pathologies during disc aging and degeneration [9]. Basal autophagy could contribute to maintaining homeostasis in disc NP notochordal cells under physiological conditions.

We previously reported the involvement of apoptosis in NP notochordal cell disappearance during disc degeneration [27,28]. The present multi-color immunofluorescence for autophagy, apoptosis, and notochordal markers further identified robust autophagy in notochordal cells of non-degenerated discs and decreased autophagy but increased apoptosis in non-notochordal cells of temporary static compression-induced degenerative discs. The pro-survival effects of autophagy are well known in diverse cell types [49], including disc cells and chondrocytes [20,21,22,23,41,42,50]. In this study, the relationship between autophagy and apoptosis is unclear, although findings suggest an additional role of autophagy in maintaining the notochordal phenotype in disc NP cells by limiting apoptosis. Future in vivo mechanistic studies of autophagy are recommended to clarify this, which will provide insight into a potential biological intervention to treat and prevent disc degeneration through autophagy modulation.

The discrepancy between in vitro and in vivo expression patterns of LC3 and p62/SQSTM1 should be noted. Unlike in vitro autophagy induction with LC3-II increases and p62/SQSTM1 decreases under serum deprivation, in vivo decreases in LC3-II and p62/SQSTM1 under static compression do not necessarily indicate autophagy deficiency. Decreased LC3-II indicates reductions in autophagosome formation and maturation [34], resulting in the loss of autophagic potential. Meanwhile, decreased p62/SQSTM1, an adaptor protein in autophagosome degradation [35], may reflect severely degenerated conditions after impaired autophagy by unphysiological mechanical loading. It is unknown whether distributed autophagy-related proteins are remnants of the notochordal phenotype [10,11]. Altered autophagy levels in response to diverse stress conditions, e.g., inflammation, during degeneration are also controversial [20,44,45]. Further investigation is required to understand in vivo autophagy under chronic stress.

The biggest limitation of this study is only the observational design. Our immunofluorescent analysis explained plausible house-keeping functions of autophagy in NP notochordal cells; however, it remains undetermined whether reduced autophagic capabilities in loaded discs result from or in degeneration. Mechanistic induction and inhibition of autophagy are subjects to be studied in the future [20]. Then, in vivo, short-term and unphysiological induction of disc degeneration in this animal model should carefully be interpreted as at least a part of human disc degeneration is the long-term natural aging process. In fact, the temporary static compression model is rather focused on the observation of chronic recovery responses from acute mechanical stress. Furthermore, in vitro, the inference from rat to human studies has severe limitations due to the presence of few NP notochordal cells and chondrocyte-like cells in human disc surgical specimens. However, autophagy in musculoskeletal disorders is not fully unveiled. This study provides the motivation to apply the disc as a good target for autophagy-modulating therapies based on its original abundance.

## 4. Materials and Methods

### 4.1. Ethics Statement

All experimental procedures were performed under the approval and guidance of the Institutional Review Board (160004, 05/16/2016 approval) and Animal Care and Use Committee (P140609, 06/19/2014 approval) at Kobe University Graduate School of Medicine. Written informed consent was obtained from each patient in accordance with the principles of the Declaration of Helsinki and the laws and regulations of Japan.

### 4.2. Antibodies and Reagents

The antibodies and apoptosis detection kit used were as follows: LC3 (3868), Cell Signaling Technology (Danvers, MA, USA); p62/SQSTM1 (ab56416) and Pax1 (ab95227), Abcam (Cambridge, UK); beclin1 (sc-11427), brachyury (sc-17743), and CD24 (sc-11406), Santa Cruz Biotechnology (Santa Cruz, CA, USA); α-tubulin (T9026), Sigma-Aldrich (St. Louis, MO, USA); TUNEL (11684795910), Roche Diagnostics (Mannheim, Germany).

The reagents used for cell culture were as follows: Dulbecco’s modified Eagle’s medium (DMEM) (D5796) and fetal bovine serum (FBS) (F2442), Sigma-Aldrich; penicillin/streptomycin (26253-84), Nakarai Tesque (Kyoto, Japan); collagenase type 2 (LS004176), Worthington Biochemical (Lakewood, NJ, USA).

The reagents used for Western blotting and immunostaining were obtained from Thermo Fisher Scientific (Waltham, MA, USA), Nakarai Tesque, Bio Craft (Tokyo, Japan), GE Healthcare (Chicago, IL, USA), Nichirei Biosciences (Tokyo, Japan), and Dako (Glostrup, Denmark).

The other reagents used were obtained from Wako Pure Chemical Industries (Osaka, Japan).

### 4.3. Patients

Six adult patients who underwent lumbar (L) and sacral (S) interbody fusion surgery because of degenerative disc disease at one L3–L4, four L4–L5, and one L5–S1 (age, 63.8 ± 12.6 (range, 44–81) years; male 3, female 3; Pfirrmann degeneration grade [43], 3.5 ± 0.5 (range, 3–4)) were enrolled in this study. Human disc tissues from discarded surgical waste were harvested for cell isolation immediately after surgery (*n* = 6).

### 4.4. Animals and Surgical Procedures

In total, 42 12-week-old male Sprague–Dawley rats (CLEA Japan, Tokyo, Japan) in weight from 435 to 509 g were used. Rats reach ≈90% skeletal maturity at 12 weeks [24,25,26,27,28]. Rat tails were affixed with an Ilizarov-type apparatus with springs, similar to that of Iatridis et al. [36], between the 8th and 10th coccygeal (C) vertebrae as described previously (Figure 2A) [24,25,26,27,28,29]. Briefly, under intraperitoneal anesthesia, two-cross 0.7 mm diameter Kirschner wires were inserted percutaneously into each vertebral body perpendicular to the tail’s axis and attached to aluminum rings. Rings were connected longitudinally with four threaded rods. Four 0.50-N/mm calibrated springs were installed over each rod. After instrumentation, axial compressive load at 1.3 MPa was applied from the distal side. Before and after surgery, all rats were fed separately in a specific pathogen-free housing cage with free access to food and water. The room was maintained on a 12 h light/dark cycle with a controlled temperature (23 ± 2 °C) and humidity (55 ± 5%).

Two rat tail experiments were designed with different loading protocols—sustained and temporary static compression. In the sustained static compression study, 24 rats were randomly loaded for 0 (unloaded for 56 days as the sham), 7, 28, or 56 days and thereafter euthanized (Figure 2B) [24,25,26,27]. While C8–C9 proximal-experimental and C11–C12 proximal-control discs were collected for Western blotting, C9–C10 distal-experimental and C12–C13 distal-control discs were applied for immunohistochemistry (*n* = 6/time point and experimental condition). In the temporary static compression study, 18 rats were randomly divided into three groups based on the loading duration and euthanized at 56 days after surgery: sham—unloaded throughout, D1—loaded for 24 h and subsequently unloaded, and D7—loaded for 7 days and subsequently unloaded (Figure 2C) [28,29]. Then, C8–C9 experimental and C11–C12 control discs were used for histomorphology and immunofluorescence (*n* = 6/experimental condition).

In six of these 12-week-old male rats, residual lumbar discs at L1–L6 vertebra were harvested for cell isolation immediately after euthanasia (*n* = 6).

### 4.5. Cells

Human and rat disc tissues were carefully dissected into the NP and AF and digested in 1% penicillin/streptomycin-supplemented DMEM with 10% FBS and 0.114% collagenase type 2 for 1 (for NP)–12 (for AF) h at 37 °C. Isolated cells were grown to ≈80% confluence in 1% penicillin/streptomycin-supplemented DMEM with 10% FBS at 37 °C under 2% O_2_ to simulate the physiologically hypoxic disc environment [15]. In a 6-well plate, 1 × 10^5^ first-passage, monolayer human and rat disc NP and AF cells/well were applied to 72 h culture in 1% penicillin/streptomycin-supplemented DMEM with 10% FBS, to 12 h pre-conditioning with 1% FBS and then to 24 h treatment of serum starvation with and without 10% FBS at 37 °C under 2% O_2_. Cells were used for protein extraction immediately after treatment (*n* = 6/species, cell type, and experimental condition).

### 4.6. Protein Extraction

Cells were placed on ice, washed, and lysed in the 3-(*N*-morpholino)propanesulfonic acid buffer supplemented with protease inhibitors. Tissues from rat caudal discs were carefully dissected into the NP and AF and homogenized using the MS-100R bead-beating disrupter (Tomy Seiko, Tokyo, Japan) for 30 s twice at 4 °C in the T-PER tissue protein extraction reagent with protease inhibitors. Soluble proteins were collected after centrifugation at 12,500× *g* for 15 min at 4 °C. Protein concentration was determined by the bicinchoninic acid protein assay and stored at −80 °C.

### 4.7. Sodium Dodecyl Sulfate (SDS)–Polyacrylamide Gel Electrophoresis (PAGE) and Western Blotting

Equal 30 µg amounts of total protein extracts were mixed with the SDS–PAGE sample buffer, boiled for 5 min, and resolved on a 7.5–15% polyacrylamide gel. Separated proteins in the tris(hydroxymethyl)aminomethane–glycine–SDS buffer system were transferred to a polyvinylidene difluoride membrane. After blocking, the membrane was incubated with primary antibodies for autophagy-related LC3 and p62/SQSTM1 (1:1000 dilution), NP notochord-related brachyury and CD24 (1:200 dilution), NP-related and AF-related Pax1 (1:1000 dilution), and loading control tubulin (1:1000 dilution) followed by incubation with peroxidase-conjugated secondary antibodies (1:200 dilution). Signals were visualized by enhanced chemiluminescence and images were taken using the LAS-3000 mini luminescent image analyzer (Fujifilm, Tokyo, Japan). Relative band intensity was quantified using the ImageJ software (http://rsbweb.nih.gov/ij/, accessed on 16 May 2016).

### 4.8. Paraffin-Embedded Tissue Preparation

Vertebral body–disc–vertebral body units in the rat caudal spine were excised, fixed en bloc in 4% paraformaldehyde, decalcified in 10% ethylenediaminetetraacetic acid, embedded in paraffin, sectioned from the mid-sagittal plane at 5-µm thickness, and prepared for histological analysis.

### 4.9. Histomorphology

Sections were stained with safranin-O, fast green, and hematoxylin or H&E. Images were obtained using the BZ-X700 microscope (Keyence, Osaka, Japan).

### 4.10. Immunohistochemistry

Sections after antigen retrieval were incubated with primary antibodies for autophagy-related beclin1 (1:40 dilution), LC3 (1:50 dilution), and p62/SQSTM1 (1:50 dilution) at 4 °C overnight, and subsequently with peroxidase-labeled secondary antibodies (1:200 dilution) at room temperature for 30 min. The blot was developed using peroxidase substrate 3,3′-diaminobenzide. Counterstaining was performed with hematoxylin. Parallel sections treated with normal IgG at equal protein concentrations were used as negative controls. The number of immunopositive cells was counted in five random high-power fields (×400) using the BZ-X700 microscope and ImageJ. Counting was performed in random duplicate sections. Positive staining was calculated as the percentage of immunopositive cells to total cell population measured by counting the nuclei.

### 4.11. Immunofluorescence

Sections after antigen retrieval were incubated with primary antibodies against brachyury for the notochordal phenotype (1:25 dilution), LC3 for autophagy (1:50 dilution), and fluorescein-labeled TUNEL for apoptosis (1:10 dilution according to the manufacturer’s instructions) at 4 °C overnight, and subsequently with Alexa Fluor 647-labed and 568-labeled antibodies (1:200 dilution) at room temperature for 2 h. Counterstaining was performed with 4′,6-diamidino-2-phenylindole (DAPI). To measure total cell number, DAPI-positive nuclei were counted in five random high-power fields (×400) of duplicate sections using the BZ-X700 microscope and ImageJ. To measure notochordal cell number, immunopositive cells for brachyury were counted similarly. Positive staining for autophagy and apoptosis was calculated as the percentage of LC3-positive cells and TUNEL-positive cells in total cells, notochordal cells, and non-notochordal cells, respectively.

### 4.12. Statistical Analysis

Data are expressed as the mean ± 95% confidence interval (CI) of six independent samples in duplicate (each *n* = 6). After evaluating the data normality by using the Shapiro–Wilk test and observing the histogram and boxplot shapes, two-way analysis of variance (ANOVA) with the Tukey–Kramer post hoc test was used to assess changes for effects of species, cell type, time point, and experimental condition. Consequently, no obvious violations of the normality assumption were found. Statistical significance was assessed with *p* < 0.050 using IBM SPSS Statistics 23.0 (SPSS, Chicago, IL, USA).

## 5. Conclusions

Experimental intervertebral disc degeneration and notochordal cell disappearance presented with decreased autophagy, a stress-response cell survival mechanism, and increased apoptosis, a programmed cell death. Further studies are warranted to have the interpretation that autophagy could protect disc NP notochordal cell homeostasis against apoptosis. Nevertheless, modulation of autophagy is a suggested more physiological molecular treatment strategy for degenerative disc disease.

## Data Availability

The data presented in this study are available on reasonable request from the corresponding author.
